# Commencements

**Published:** 1898-07

**Authors:** 


### COMMENCEMENTS.

### Cincinnati College of Dental Surgery.

The annual commencement exercises of the Cincinnati College of Dental Surgery were held in the Auditorium, Cincinnati, Ohio, on Tuesday evening, April 12, 1898.

The annual address was delivered by Professor G. S. Junkerman, M.D., D.D.S., dean of the college, and the valedictory by Rev. Charles F. Goss.

The number of matriculates for the session was eighty-nine.

The degree of D.D.S. was conferred on the following graduates by Hon. Francis B. James, L.L.B., president of the board of trustees:

| NAME. | STATE OR COUNTRY. | NAME. | STATE OR COUNTRY. |
|---|---|---|---|
| R T Cisler | Ohio | Hanna Hurvitz | Russia |
| D G Davidson | West Virginia | Walter C Jones | Ohio |
| Harry L Davis | Ohio | Chas T Meyer | Ohio |
| W F Eagleson | Ohio | Harry C Murphy | Indiana |
| John H Francis | Ohio | Lucian Q Nelson | Kentucky |
| Robert J Hanran | Canada | John Pfannstiel | Ohio |
| Oscar S Heineman | Ohio | Albert C Stamm | Ohio |
| Elmer B Hosom | Ohio | John R Smyth | Indiana |
| Herman B Hune | Ohio | I A Winston | Ohio |

### Detroit College of Medicine.

The Annual Commencement exercises of the department of dental surgery, Detroit College of Medicine, was held in Harmonic Hall, Thursday, June 16, 1898.

Rev. N. Boynton opened the exercises by prayer. Commencement address was delivered by W. C. Barrett, M.D., D.D.S., of Buffalo.

The address to the graduates was delivered by T. J. Collins, D.D.S. The degree of D.D.S. was conferred upon the following candidates:

| NAME. | RESIDENCE. | NAME. | RESIDENCE. |
|---|---|---|---|
| J H Armstrong | Ontario | H Iler | Ontario |
| J H Blain | Michigan | C M Leonard | Michigan |
| W A Brown | Ontario | D A McMillan | Ontario |
| S Bump | Michigan | J W McVicar | Ontario |
| C E Cochrane | Michigan | C C Messenger | Michigan |
| L M Dickens | Michigan | W W Mitchell | Michigan |
| K M Fechheimer | Michigan | D M Simpson | Ontario |
| D M Graham | Ontario | D B Tower | New Hampshire |
| C J Gray | England | H K Weber | Ontario |
| G Hackett | Ontario | C E Whitmore | Michigan |
| G L Haywood | Michigan | E Wicks | Ontario |
| E Higgins | Michigan | G Wolcott | Michigan |
| S Hodgson | Ontario | | |

### Marion-Sims College of Medicine, Dental Department.

The annual commencement exercises of the Dental Department of the Marion-Sims College of Medicine were held in the Fourteenth Street Theatre, St. Louis, Mo., on Saturday evening, April 9, 1898.

The address to the graduates was delivered by Dr. Theodore Menges.

The number of matriculates for the session was seventy-five.

The degree of D.D.S. was conferred on the following graduates by Y. H. Bond, M.D., dean of the college.

| NAME. | RESIDENCE. | NAME. | RESIDENCE. |
|---|---|---|---|
| Samuel T Bassett | Missouri | Walter Hopkinson | Illinois |
| Edmund N Beall | Missouri | Lawrence W Mrazek | Missouri |
| Herman H Born, M.D | Missouri | David Osterheld, Jr | Missouri |
| Oscar L Bumiller | Missouri | Richard L Parrish | Missouri |
| Wm L Cripliver | Missouri | Aarold G Powell | Illinois |
| Chas H Downing | Illinois | Harry A Rathbun | Kansas |
| Alvin Godejohann | Illinois | Jos C Reader | Illinois |
| Roland B Harvey | Kansas | Frank LeR Tucker | Illinois |

### Ohio College of Dental Surgery.

The fifty-second annnal commencement exercises of the Ohio College of Dental Surgery (Department of Dentistsy of the University of Cincinnati) were held at the Odeon, Cincinnati, Ohio, Thursday evening, April 5, 1898.

The annual address was delivered by Dr. W. O. Thompson, president of Miami University, and the class address by Charles E. Cook, D.D.S.

The number of matriculates was two hundred and six.

The degree of D.D.S. was confered on the following graduates by Dr. D. W. Clancy, vice-president of the board of trustees :

| NAME. | RESIDENCE. |
|---|---|
| James H Arbuckle | Ohio |
| John K Bannister | Kentncky |
| Adelbert W Beard | Ohio |
| Irvin R Benner | Ohio |
| Adalbert B Binns | Georgia |
| W E Blackburn | Ohio |
| Eugene L Blazer | West Virginia |
| John W Bonzo | Ohio |
| Arthur Y Boucher | Ohio |
| Ernest E Branch | Ohio |
| Homer M Brewer | Ohio |
| H A Broering | Ohio |
| W B Cadwell | Ohio |
| Charles E Cook | Ohio |
| Charles F Cooper | Ohio |
| Stanley M Copland | Michigan |
| Herbert S Curtis | Ohio |
| Edw M Donahue | Iowa |
| Fred W Everist | Ohio |
| Frank B Ford | Ohio |
| T W Forshee, Jr | Indiana |
| Earl Gear | Indiana |
| Robert L Graves | Kentucky |
| E L Griffin | Ohio |
| Eugene I Harlan | Ohio |
| M D Hartinger | Ohio |
| John B Hodge | Ohio |
| Charles O Hook | Ohio |
| Walter E Jenny | Ohio |
| Edw T Johnson | Ohio |
| Miss F L Kaplan | Ohio |
| Edward Kelley | Ohio |
| Lewis C King | Michigan |

| NAME. | RESIDENCE. |
|---|---|
| Alfred J Koelble | Ohio |
| L B Longenecker | Ohio |
| H K McCulloch | Pennsylvania |
| Frank McLean | Ohio |
| Roy B Meek | Ohio |
| William R Meeker | Indiana |
| Edward H Meyer | Indian0 |
| Jno R Molyneaux | Ohio |
| Elmor E Morris | Ohio |
| Marion E Myers | Ohio |
| William M Myerr | Kentucky |
| Robert C Pollitt | Kentucky |
| Joseph W Pritchard | Indiana |
| Joseph M Ratliff | Ohio |
| William C Rice | Illinois |
| Ottis P Robinson | Indiana |
| Jacob W Rohrer | Ohio |
| Herman E Rose | Indiana |
| Ira H Scott | Ohio |
| William H Sells | Ohio |
| Rudolqh Siegel | Ohio |
| L G Singleton | Pennsylvania |
| John S Stewart | Indiana |
| S W Taliaferro | Kentucky |
| George F Thomas | Ohio |
| O A Thompson | Ohio |
| Guy B Tuthill | Michigan |
| Charles V Vicroy | Kentucky |
| Ceo H Williamson | Ohio |
| Charles W Wilson | Michigan |
| Cary A Wisecup | Ohio |
| Wier M Wood | Pennsylvania |
| Z N Wright | Ohio |

### University of Michigan, Dental Department.

The Annual Commencement exercises of this department was held in connection with that of all other departments of the University, on Thursday, June 30, 1898.

The University Hall, which holds about 4,000 people, was filled to its utmost capacity.

The exercises were opened by music and prayer. The Commencement oration was delivered by Benjamin Ide Wheeler, L.L. D., of Cornell University.

The degree of Doctor of Dental Surgery was conferred upon the persons named below, fifty-three in number.

The whole number in attendance in this department was 223. The total number in attendance at the University for the year, 3,223.

RESIDENT GRADUATES.

| NAME. | RESIDENCE. | NAME. | RESIDENCE. |
|---|---|---|---|
| Frederick John Howard Leland, D.D.S., University of Tennessee | Saint Johns | Benjamin Wolf, D.D.S., Ohio Dental College | Cincinnati, O |

SENIORS.

| NAME. | RESIDENCE. | NAME. | RESIDENCE. |
|---|---|---|---|
| Roy Archbold | Decatur, Ind | Chalmers J Lyons | Mt Pleasant |
| Alfred Baldwin | Providence, R I | Harry B McMillan | Grand Rapids |
| Richard E Bloomer | Keithsburg, Ill | Edwin K Medler | Sault Ste Marie |
| Walter Herbert Bowman | Toledo, O | Stephen A D Merchant | Harlan, Ind |
| Lyman Smith Brown | Hudson | Fred Colonel Miller | Hammond, Wis |
| Francis Charles Castell | Pontiac | James Weston Minerd | Battle Creek |
| W Alonzo Chamberlain | Muskegon | Guy Raymond Palmer | Charlotte |
| John Francis Conley | Dexter | Leslie Ward Platt | Niles |
| J. Trower Davies, M.D | Battle Creek | Lester George Platt | Niles |
| James Roy Davis | Ann Arbor | Thomas Crompton Reid | Lapeer |
| James Barnard Doyle | Grand Rapids | John Martin Rich | Haricon, Wis |
| R Norman Forbes | Centre Lisle, N Y | Claude Burus Roe | Buchanan |
| Percy Robert Glass | Cadillac | Ralph Jay Roper | Santa Ana, Cal |
| Claude Charles Goodes | Flint | John Henry Setzler | Vandalia |
| Richard Bertram Hamilton | Lima, O. | Arthur B Snow | Saginaw, West Side |
| Milton J Hardy | Austin, Minn | Joseph B Stewart | New York, N Y |
| Harold Martin Herron | Ann Arbor | John Howard Stofflet | Vicksburg |
| R Brown Howell | Ann Arbor | Arthur M Sweet | Providence, R I |
| Bessie Hutchinson | Ann Arbor | Daniel Michael Thompson | Detroit |
| Ulysses Simpson Jeffs | Rockland | Fred Giles Titus | Montevideo, Minn |
| G Norman Kimball | Pt. Gamble, Wash | Thomas Budd Van Horn | Franklin, O |
| Alexander H. Kinmond | Saint Johns | Harry Melville Viel | Fenton |
| Marc Anthony Kroupa | Traverse City | B Franklin Vosburgh | Ann Arbor |
| Carl Hans Lebert | Stuttgart, Germany | Philip Ernest Waugh | Cedar Falls, Ia |
| Herbert Edgar Lehr | Marine, Ill | Oliver Wilson White | Chatham, Ont |
| James McM Loudon | Traverse City | Lewis Denison Zincke | Chelsea |

The degree of Doctor of Dental Science was also conferred upon T. E. Carmody, D.D.S., Owosso; and Dessie B. Robertson, D.D.S., McConnelsville, O.; making fifty-five in all.

### Northwestern University Dental School.

The annual commencement exercises of the Northwestern University Dental School were held in the Grand Opera House, Chicago, Ill., on Tuesday afternoon, April 5, 1898.

The salutatory address was delivered by Mr. John L. Wagner, the doctorate address by W. T. Eckley, M.D., and the valedictory by Charles A. Young, D.D.S.

The degree of D.D.S. was conferred on the following graduates by Henry Wade Rogers, LL.D., president of the university:

Holson Allen

Herbert E Algeo

Edwin J Albrecht

Robert T Aiston

Clarence A Atwood

George B Atchinson

Louis E Badgley

Fred P Barnhart

Walter A Brown

George C Brandt

Arthur S. Bayne

Henry C Brock

Clara F Brundage

G H Bridgman, D.D.S.

Frank E Burnett

Louis E Blackaller

Alexander J Bell

George H Beidler

Frank H Bimrose

Clara C Beck

John E Burgan

Claude P Banning

Arthur W Beach

Thomas J Boyce

Samuel B Crawford

Harry Copley

Frank Curtis

Robert I Coles

John C Conniff

Charles M Clough

Oscar Carrabine

George W Darnell

James C Davis

Benjamin F Dowell

Leslie G Derrick

Walter F Dorsey

Walter K Delano

Ulysses F DeVries

Clyde S Demorest

Lewis M Ellis

Herman W Elliott

William C Eustice

Harry F Echternacht

Lewis C Edison

Carroll R Eaton

Herman L Frankel

Roger L Fellows

William F Froeschle

Frank N Freemire

Van E Freeman

John H Fowler

Albert M Ford

Carlton M Garrett

Nathaniel L Garling

Willis M Graham

William R Gibson

Arthur C Grotewohl

Frederick Graflund

Harry Gulmyer

James Grove

Charles H Godward

Francis Hayes

William J Hopf

Alice C Hunter

Charles B Hess

Francis E Heacox

Rudolph G Hunn

Clemens Huberty

Geo H Hillenbrand

Walter L Hollenbeck

Clark G Hibbard

Turner B Hollenbeck

Almont C Hixon

James H Haight

Arthur V Hargett

Norman H Hunter

Harry Hoare

Clark K Hartzell

Lloyd R Hawley

Harry R Isenhower

Arthur L Inglesby

Glyde W Johnson

Walter W Johnson

J DeWolf Jackson

Walter H Jackson

John R Jones

Sherman Keyser

Gertrude Kniewel

Conrad F Krenwinkel

John H Kennedy

Chas E Klopp

Ray D Kellogg

Chas H Lawson

Charles S Leininger

Olof Lange

Frederick S Lawrence

James J Leonard

Ralph E Libberton

Earnest J Morgan

James W Madden

Frank M Metzgar

Geo W Minium

Burt M Mott

Shane A Morgan

Royal B Munn

Theodore H Mentague

Ebbin A Meservey

William E Morris

Samuel W McNinch

Frank E McDermott

John McIntyre

Robert P Neil

Joseph J Onthank

Wiley L Overholser

Katie D Prothero

Mary Peak

Houghton Peck

Geo M Perry

Walter S Padget

Ross T Parshall

Emerson J Prettyman

Raymond C Quick

Homer E Robinson

Geo M Russell

Fred T Rench

Chas N Reese

William F Rimes

Milo E Radliff

George R Richardson

Fred A Reisacher

Harry R Risinger

Theodore H Rath

Edward F Rowell

Guy M Ruff

Fletcher C Rood

Isaac E Reid

Forrest D Reed

James M Snow

Harry L Shupert

Aaron J Shepherd

L Langdon Sheffield

Charles Schlotfeldt

Dan P Scott

Madge L Schaum

Jas H Shields, D.D.S.

Lucien W Strong

Wm A Stephenson

John J Sulberger

Chas W Stoufer

Wilbert S Sweet

Walter C Taylor

John T Taylor

Arthur C Tucker

Clarence C Thomas

Henry G Trent

Burton M Tunison

John VanDyke

Charles D Whitcomb

Geo W Wilson

Guy F Wait

Chas R Wheeler

John L Wagner

Miles E Walton

Harry C Wood

Chas S Wicklin

Franklin F Wing

Thomas P Wise

James B Watts

Daniel W Wickham

Samuel E Wallace

Clarence A Webb

John H Wallace

Frederick V Williams

Harry K Williams

Thos B S Wallace

Chas A Young

### University of Pennsylvania Dental Department.

The Annual Commencement exercises of this institution was held June 8th, 1898, in the Academy of Music.

The exercises of the occasion were opened with prayer by

Rev. Dr. Spaeth, after which a hymn "Our Father in Heaven" was sung. The Commencement address was delivered by Judge J. B. McPherson.

The number in attendance during the session was two hundred.

The degree of D.D.S. was conferred upon the following candidates, graduates of the University of Pennsylvania, June 8, 1898:

Warren Babcock

Charles H Bankhead

Minot V Bastian

Horace P Beck

Thomas C Beswick

William W Booth

Charles L Brininstool

Rudolph Bum

Jacob T Butz

Henri Pierre Calais

Alonzo C Caldwell

Noble C Campbell

Karl E Carlson

Raymond F Casler

William R Chaplin

Joseph W Cheyney

James T Coyle

William L Cramer

Edward G Cunningham

Michael J Danahy

Charles F DuFour

Charles C Dupont

Richard L Entwistle

John Erwin

Joseph C Farmer

Warran VanH Filkins

Walter H Fordham

Floyd C Frederick

Daniel L Gallivan

William Gardner

John H Gartrell

Albert N Gaylord

Paul E Gies

Hans J V Gruter

Joshua G Hagee

George G Hansell

Hugo C Hark

James M Hastings, Jr

John I Hay

Clare M Henry

James K Hunter

Zachary T Jackaway

Hiram C Jacobs

Marshall J A James

Edward M Jones

Paul C Jones

William L Keller

Joseph Kussy

Samuel E Langfitt

Eugene A Lincoln

Oliver G Longenecker

Roy P McCowan

David McGrowan, Jr

Frederick E McLaren

Valentine Macdonald

Otto I Metzger

John D Millikin

Carl T Moehring

Tolbert B Moorhead

Clyde M Naley

Henry C Newkirk

Charles H O'Neill

Byron W Palmer

Charles C Payne

George Parry

John W Parsons

Leroy E Perkins

Taliesin Phillips

William D Pursel

David J Reese

Alexander H Reynolds

Walter Richards

Franklin B Roberts

Edwin W Robinson

Alfred H Sage

Frederick H Saunders

Alfred R Schermerhorn

John G Sears

Louis R Seymour

Patrick W Shields

Donald H Shoemaker

Alfred H Sinsabaugh

Samuel S Smith

John Stearns

Marcus R Stewart

Leon B Tantum

Charles R Turner

Ralph W Waddell

Thomas B Wade

Hans T Waltisbuhl

George B Weakley

Walter Webb

Fred F Wood

Arthur W Woodcock

Howard H Woodrow

### Commencement University of Minnesota.

The graduation exercises of the University of Minnesota Dental Department was held Thursday June 2nd, 10 A.M. in the Armory Hall. After the usual exercises the degree of D.D.S. was conferred upon the following candidates:

Almon G Atwood

Chas A Couplin

John Jeffers

Rolf J Olson

Wm E Spencer

Sidney E Bennion

Eugene C Fischer

John R Lawton

Fred W Prail

E James Van Bronkhorst

Earnest Avery Wright

Wm H Card

J Roy Hollister

C Newhouse

Edward H Shibley

Fred K Weible

### Kansas City Dental College.

The sixteenth annual commencement exercises of the Kansas City Dental College were held in the Coates Opera House, Kansas City, Mo., on Tuesday, April 5, 1898.

The faculty address was delivered by Professor R. T. Sloan, A.M., M.D., and the salutatory by T. M. Cockrill, D.D.S.

The degree of D.D.S. was conferred on the following graduates by A. H. Thompson, D.D.S., president of the faculty:

| NAME. | STATE. | NAME. | STATE. |
|---|---|---|---|
| Adelbert D Ames | New York | Wm B McCrary | Kansas |
| Geo A Baker | Kansas | Tracy M Payfair | Missouri |
| Thos M Cockrill | Missouri | Thos B Ramsey | Missouri |
| Francis M Caldwell | California | Earl W Shrock | Missouri |
| O O S Charlston | Kansas | W E Schumann | California |
| Wm E Dodds | South Dakota | Richard M Seibel | Missouri |
| John A Dampf | Missouri | W D Shackelford | Texas |
| Geo A Esterly | Ohio | Jas E Shewmaker | Missouri |
| Benj L Foster | Illinois | Henry M Waters | Iowa |
| Frank A Glasscock | California | Geo W Ward | Kansas |
| Earl A Henderlider | Kansas | Styles W Wherry | Kansas |
| James E Hardy | Kansas | Benedict G. Wenker | Missouri |
| Emil M Kapi'an | Wisconsin | Edouardo P Wheeler | Kansas |
| Andrew F Kerns | Kansas | Dixon H Woods | Kansas |
| Ed A Miller | Missouri | Fred M Yingling | Kansas |
| Edw T McDowell | Missouri | John C Yager | Ohio |

### List of Committees for the Joint Meeting of the Southern Branch of the National Dental Association and the Louisiana State Dental Society.

No. 1—Committee on Arrangements, in subdivisions, Transportation Committee: Dr. Joseph Bauer, (S. B. N. D. A.), chairman, New Orleans, La. Hotels and Quarters Committee: Dr. J. Rollo Knapp, (S. B. N. D. A.), chairman, New Orleans, La. Hall, Exhibits and Arrangements Committee: Dr. L. D. Archinard, (L. S. D. S.), chairman, New Orleans, La.

No. 2—Committee on Clinics: Dr. T. P. Hinman, (S B. N. D. A.), chairman, Atlanta Ga. Committee on Clinics, Louisiana State Dental Society: Dr. And. G. Friedrichs, (L. S. D. S.), chairman, New Orleans, La.

No. 3—Committee on Publication: Dr. Shep. W. Foster, (S. B. N. D. A.), chairman, ex-officio, Atlanta, Ga.

No. 4—Committee on Oral Hygiene: Dr. L. M. Cowardin, chairman, Richmond, Va.

No. 5—Committee on Prosthetic Dentistry: Dr. J. A. Dale, chairman, Nashville, Tenn.

No. 6—Committee on Orthodontia and Oral Surgery: Dr. Geo. Hardy, chairman, Baltimore, Md.

No. 7—Committee on Operative Dentistry: Dr. J. G. Fife, chairman, Dallas, Texas.

No. 8—Committee on Chemistry, Metallurgy and Anatomy: Dr. Albert B. King, chairman, Baltimore, Md.

No. 9—Committee on Pathology, Materia Medica and Therapeutics: Dr. H H. Johnson, chairman, Macon, Ga.

No. 10—Committee on Microscopy, Histology and Bacteriology: Dr. W. T. Martin, chairman, Yazoo City, Miss.

No. 11—Committee on Appliances and Improvements: Dr. T. M. Allen, chairman, Birmingham, Ala.

No. 12—Committee on Education, Literature and Voluntary and Special Essays: Dr. T. P. Whitby, chairman, Selma, Ala.

Entertainment Committee: Dr. Joseph Bauer, (L. S. D. S.), chairman, New Orleans, La.

Carnival Balls Invitations Committee: Dr. J. Rollo Knapp, (S. B. N. D. A.), chairman, New Orleans, La.

WM. E. WALKER, *Pres. Christian Pass, Miss.*

C. L. ALEXANDER, *Cor. Sec'y. Southern Branch National Dental Association.*

CHARLOTTE, N. C., July 12, 1898.

